# Investigations on the Surface Integrity and Wear Mechanisms of TiAlYN-Coated Tools in Inconel 718 Milling Operations

**DOI:** 10.3390/ma17020443

**Published:** 2024-01-17

**Authors:** Francisco J. G. Silva, Naiara P. V. Sebbe, Rúben D. F. S. Costa, André F. V. Pedroso, Rita C. M. Sales-Contini, Marta L. S. Barbosa, Rui P. Martinho

**Affiliations:** 1 ISEP, Polytechnic Institute of Porto, Rua Dr. António Bernardino de Almeida, 4249-015 Porto, Portugal; napvs@isep.ipp.pt (N.P.V.S.); afvpe@isep.ipp.pt (A.F.V.P.); rita.sales@fatec.sp.gov.br (R.C.M.S.-C.); rpm@isep.ipp.pt (R.P.M.); 2 Associate Laboratory for Energy, Transports and Aerospace (LAETA-INEGI), Rua Dr. Roberto Frias, 400, 4200-465 Porto, Portugal; rdcosta@inegi.up.pt; 3 Department of Mechanical Engineering, Faculty of Engineering, University of Porto, Rua Dr. Roberto Frias, 400, 4200-465 Porto, Portugal; martabarbosa8c@gmail.com; 4 Technological College of São José dos Campos, Centro Paula Souza, Avenida Cesare Mansueto Giulio Lattes, 1350 Distrito Eugênio de Melo, São José dos Campos 12247-014, Brazil

**Keywords:** milling, Inconel 718, TiAlYN coatings, HiPIMS technique, tool wear mechanisms, surface integrity

## Abstract

Inconel 718 is a Ni superalloy with superior mechanical properties, even at high temperatures. However, due to its high hardness and low thermal conductivity, it is considered a difficult-to-machine material. This material is widely used in applications that require good dimensional stability, making the milling process the most used in machining this alloy. The wear resulting from this process and the quality of the machined surface are still challenging factors when it comes to Inconel 718. TiAlN-based coating has been used on cutting tools with Yttrium as a doping element to improve the process performance. Based on this, this work evaluated the machined surface integrity and wear resistance of cutting tools coated using Physical Vapor Deposition (PVD) HiPIMS with TiAlYN in the end milling of Inconel 718, varying the process parameters such as cutting speed (*v*_c_), feed per tooth (*f*_z_), and cutting length (*L*_cut_). It was verified that the *L*_cut_ is the parameter that exerts the most significant influence since, even at small distances, Inconel 718 already generates high tool wear (TW). Furthermore, the main wear mechanisms were abrasive and adhesive wear, with the development of a built-up edge (BUE) under a125 m/min feed rate (*f*) and a *L*_cut_ = 15 m. Chipping, cracking, and delamination of the coating were also observed, indicating a lack of adhesion between the coating and the substrate, suggesting the need for a good interlayer or the adjustment of the PVD parameters.

## 1. Introduction

The class of materials known as Inconel are Ni-Cr-based superalloys, recognised for having superior mechanical properties and good fatigue and creep behaviour up to 700 °C [1]. In their composition, generally, some elements, such as Al, Ti, Nb, Co, Cu, W, and Fe, are added, with the aim to improve their mechanical properties and corrosion resistance [2]. Within this class, Inconel 718 stands out. This material is a precipitation-hardened superalloy, with elements such as Ni and Cr contributing to its corrosion resistance [3,4]. Furthermore, Inconel 718 combines its resistance to corrosion [4] with excellent mechanical properties at high temperatures and good weldability [5], and is widely used in aircraft, gas turbines, turbocharger rotors, nuclear reactors, liquid fuel rockets, critical rotating parts, airfoils, etc. [6,7,8,9]. It can represent 30% of the total weight of an aircraft engine [10,11].

However, due to its properties, such as high hardness and low thermal conductivity, conventional machining and forming processes are challenging, making this alloy a difficult-to-machine material [12,13,14]. During machining, the work hardening of this alloy and its reactivity with the cutting tool material at high temperatures plastically deform the cutting tool, resulting in an inferior surface quality of the machined part [15]. This fact, added to the tendency of this alloy to adhere to the surface of cutting tools, makes machining even more difficult [16]. However, machining is still widely used in industries to produce high-precision and quality parts [17]. Of the machining processes, milling, a more flexible process with great dimensional accuracy, is the most used in the machining of Inconel 718.

Many authors have based their research on milling Inconel 718 [18,19,20,21,22,23], emphasising the fact that it is a challenging material when it comes to conventional machining processes. For example, Liao et al. [24] analysed the end milling of Inconel 718 under various cutting speeds (*v*_c_) with carbide tools. It was found that, at low speeds, the increase in cutting temperature and strain hardening were the main problems generated in the slot milling of this alloy, causing chipping of the cutting tool and its subsequent failure. When milling at medium speeds, there was a reduction in the cutting force due to the softening caused by the precipitation γ′ of Inconel 718. However, the chips were welded when the *v*_c_ was further increased, and their flow changed. With this, the authors observed an optimal *v*_c_ range for the end milling of Inconel 718.

On the other hand, Mayiar et al. [25] optimised end milling parameters, such as *v*_c_, feed rate, and the depth of cut, when milling Inconel 718. The authors carried out nine tests on an L9 orthogonal arrangement of the Taguchi method. The analysis was based on surface roughness (SR) and the material removal rate (MRR), and an analysis of variance (ANOVA) was also applied to identify the most significant factor in the process. Based on the results, it was verified that the ideal cutting parameters would be 75 m/min, 0.06 mm/tooth and 0.4 mm for cutting depth, with *v*_c_ being the parameter with the most significant influence on the milling process. Therefore, to optimise the process, improve the tool life, reduce wear and ensure good surface integrity, machining parameters must be correctly selected, as well as the cutting conditions, environment, and the choice of the cutting tools’ materials [15]. Thus, research involving machining is still a hot topic today, and efforts are being oriented toward improving the machining process, especially regarding materials that are difficult to machine [26].

Thus, analysing recent studies and the factors involved in the milling of Inconel 718, the set of ideal cutting parameters can be predicted. These studies are essential not only for Inconel 718 but also for all processing and milling operations involving difficult-to-machine materials. Furthermore, the cutting forces developed in the process are also important and must be considered [27], as they offer information about the performance and stability of milling and, consequently, are related to wear and surface integrity [28].

One of the main problems faced during the milling of Inconel 718 is the rapid wear of cutting tools, as critical shear and temperature forces are generated during the process, which leads to premature tool failure [29]. Furthermore, the cutting tool directly impacts the process, which means that much research is based on creating new geometries for these tools [30] and the development of coatings capable of improving the process performance. Furthermore, ultrasonic vibration can also be used to reduce the wear that tools are exposed to [31]. The hardness values of the coatings, low friction coefficients, and thermal specifications directly influence the process performance [32]. These coatings can be applied to various surfaces for different industries, as well as used in injection moulds [33,34]. In this scenario, when comparing the performance and efficiency of the process, recent works have made their analyses based on the comparison between coated and uncoated cutting tools in the machining of Inconel 718, as is the case of the work by Ucun et al. [35], in which the effect of a coating material on tool wear (TW) was analysed during the milling of Inconel 718, with uncoated and DLC-coated tools (WC-Co), while changing the feed rates and cutting depths. These authors found that the use of the coating improved the SR and reduced the formation of burrs and the built-up edge (BUE), making the process performance better than when using uncoated tools.

The coatings are typically produced by two different processes, called Chemical Vapor Deposition (CVD) and Physical Vapor Deposition (PVD) [36]. These two processes include specific techniques. For example, PVD can be divided into two main processes: evaporation and sputtering [37], related to how particles can be extracted from the target. Sputtering is used more in applications that require a good surface quality [38] and has assumed extreme importance within the PVD deposition process group, with the development of new technologies and processes that aim to generate coatings with better mechanical properties, as is the case with the PVD HiPIMS (High Power Impulse Magnetron Sputtering) process. In the HiPIMS process, coatings with residual compressive stress are generated [39,40,41], which is extremely important in the milling process [42], as these stresses provide greater resistance to the tool edge, and, as a result, the quality of the machined surface is better.

However, much progress needs to be made in this area, as this can make the edges of cutting tools more susceptible to wear, which means that there are still many challenges regarding how to improve wear resistance with the use of different coatings when milling Inconel 718. The TiAlN-based coating is widely used and performs well in high-speed machining, with excellent oxidation resistance [43]. In addition, TiAlN-based coatings feature high hardness and good thermal stability [44]. However, wear resistance is still an area of much study and research. In this context, aiming to improve the tool’s performance, doping elements have been added to TiAlN [45,46,47,48]. In addition to improving wear resistance, these elements can enhance corrosion resistance, hardness, adhesion, and toughness [17]. One element that can be used as a doping element is Yttrium [49]. The importance of adding Yttrium to the TiAlN-based coating is related to improved mechanical properties and oxidation resistance [50]. This phenomenon occurs due to the segregation of the element through grain refinement during film growth and its ability to form a protective film due to Yttrium’s strong affinity for oxygen [51]. Aninat et al. [52] found that adding Y generated greater hardness and better mechanical properties; however, it reduced the compressive stress. In turn, Moser et al. [53] analysed the thermal stability of the Ti_1−x_Al_x_N coating with the addition of Y through the DC magnetron sputtering process and found that at higher temperatures, after annealing, there was an increase in hardness. Furthermore, through the characterisation and morphology generated, it was seen that Y slows down the decomposition process of supersaturated phases. Even in studies regarding the characteristics and behaviour of the TiAlYN coating, there is still a significant gap in the literature regarding its wear behaviour and performance during the machining process, with most works having focused on its characterization.

Furthermore, there are still many challenges regarding the machining of Inconel 718, specifically the milling process, as many studies only address turning [54]. Even with coatings, the wear generated in the machining process is still a topic that should be further explored. Much information can be taken from models and simulations that can predict wear behaviour [55], as well as the quality of the machined surface, quickly and economically [56]. However, these models are complex and depend on prior knowledge, in addition to being little explored in milling, especially for materials that are difficult to machine, such as Inconel 718. The wear occurring during the milling process can provide data on the productivity of the process, the need to adjust the machining parameters, the materials involved, and their interaction and affinity to understand whether they are correct or whether an adjustment is necessary [26].

Therefore, this work evaluates the influence of machining parameters, such as *v*_c_, feed per tooth (*f*_z_), and cutting length (*L*_cut_), on the surface integrity and wear behaviour of end mills coated with TiAlYN through the PVD HiPIMS process during the machining of Inconel 718. Therefore, this work aims to fill the gap regarding the use of Yttrium as a doping element and provide insight into the milling of Inconel 718.

## 2. Materials and Methods

This section will describe the materials used in the experimental work and the equipment used to perform the analyses.

### 2.1. Materials

#### 2.1.1. Workpiece Material

The workpiece material was made of Inconel 718, an austenitic Ni-Cr-based superalloy. This material was supplied as a round bar with a 158 mm diameter (*Ø*), and prepared to a length of 30 mm for carrying out the tests. It underwent the following heat treatments:

- Solution annealing at 970 °C, followed by quenching in water;
- Precipitation hardening at 718 °C for 8 h, oven cooling at 621 °C for 8 h, and air cooling.

This workpiece was purchased from the company Paris Saint-Denis Aéro (Grândola, Portugal). The material’s mechanical properties are presented in Table 1, and its respective chemical composition (%wt) is shown in Table 2.

#### 2.1.2. Substrate and Tool Geometry

The employed tools are end mills. The substrate of the tools is a cemented carbide WC-Co, grade 6110, with Cobalt (~6 wt%) as a binder and an average grain size of 0.3 μm. These tools were provided by INOVATOOLS, S.A. (Leiria, Portugal). The tool geometry is characterised in Table 3.

### 2.2. Methods

#### 2.2.1. PVD Coating

Before coating deposition, the cutting tools were cleaned with acetone in an ultrasonic bath. This cleaning was performed in two phases: the first lasted around 15 min, and then the acetone was renewed before the second cleaning phase, which lasted 5 min.

A TiAlYN coating thickness of 2.4 μm was deposited via the PVD HiPIMS process using CemeCom CC800/HiPIMS equipment (CemeCon, AG, Wuersele, Germany) with four target holders. The adopted deposition parameters can be observed in Table 4. These parameters were selected from successful previous experiments on similar substrates using different targets. The rotation speed applied for the substrate holder was 1 rpm, ensuring that the deposited coatings presented high homogeneity throughout the deposition process.

#### 2.2.2. Machining Parts

Machining tests were performed using CNC machining centre HAAS VF-2 (H.A.A.S. Automation, Oxnard, CA, USA), with three axes to machine, a maximum speed of 10,000 rpm, and a maximum power (*P*_in_) of 20 kW. A spiral milling strategy was chosen as the part was supplied with a circular geometry. Thus, milling occurred from the centre towards the periphery of the workpiece and tests were conducted using cutting fluid with 5% oil in water, Alusol SL 61 XBB, which is a semi-synthetic metalworking fluid.

**Figure 1:**
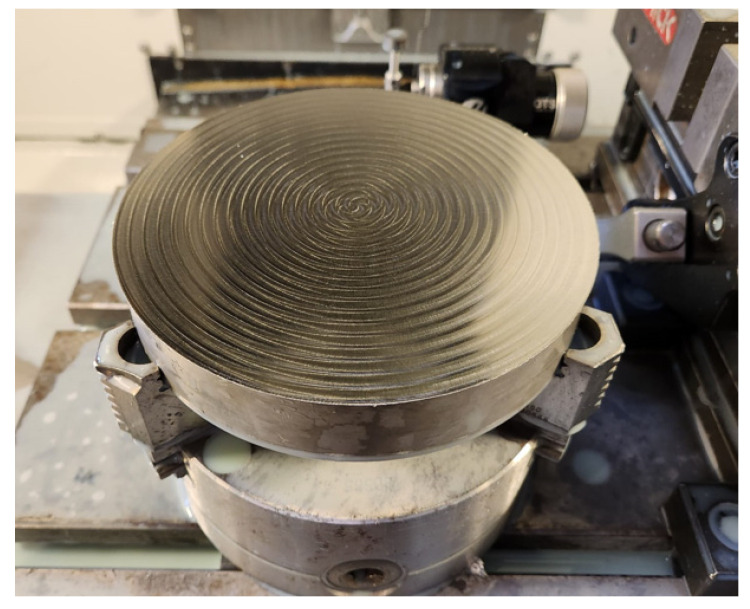
Workpiece material and its spiral marks.

Regarding the milling parameters, because the strategy chosen was a spiral to avoid wear-related phenomena, the radial depth of the cut was kept constant. Another parameter was the axial depth of the cut (*a*_p_, or ADOC). This parameter was suggested by the cutting tool supplier, therefore, initially, values of 0.2 mm and 0.1 mm were applied. However, these values caused the tool to fail and break shortly after the initial plunge. Due to this, the value of 0.08 mm was defined for this parameter, which was kept constant for all tests, as finishing milling operations was the goal to simulate. In addition, as the tool *Ø* = 6 mm, the value of the radial depth of cut (*a*_e_ or RDOC) was defined considering 75% of this value, i.e., 4.5 mm. This parameter also remained constant for all conditions tested. The parameters *v*_c_, *f*_z_, and *L*_cut_ were varied, determined based on the provided substrate of the tool. Regarding *f*_z_, the centre value (100%) was 0.0700 mm/tooth and varied by 25% for less and 50% for more. This parameter was varied as it is known to have a high impact on wear and influence the quality of the machined surface. For *L*_cut_, values of 5 m and 15 m were selected, aiming to analyse the progression of wear throughout the machining of the workpiece. For *v*_c_, values of 75, 100, and 125 m/min were used, with the purpose of comparing and analysing the influence of cutting speed on the resulting wear and surface integrity. All parameters and test conditions are shown in Table 5, and Figure 1 illustrates the workpiece with its corresponding spiral marks. To fix this workpiece, a self-centring bushing with three jaws, Bison 3575 (BISON-BIAL, Bliesk Podiaski, Poland), was used, and the tools were fixed with an ISO40 DIN69871 cone, an ER32 H70 collet holder, an ISO 7388-2 tie rod, and an ER DIN 6499 collet from the same manufacturer.

#### 2.2.3. Machined SR Analysis

**Figure 2:**
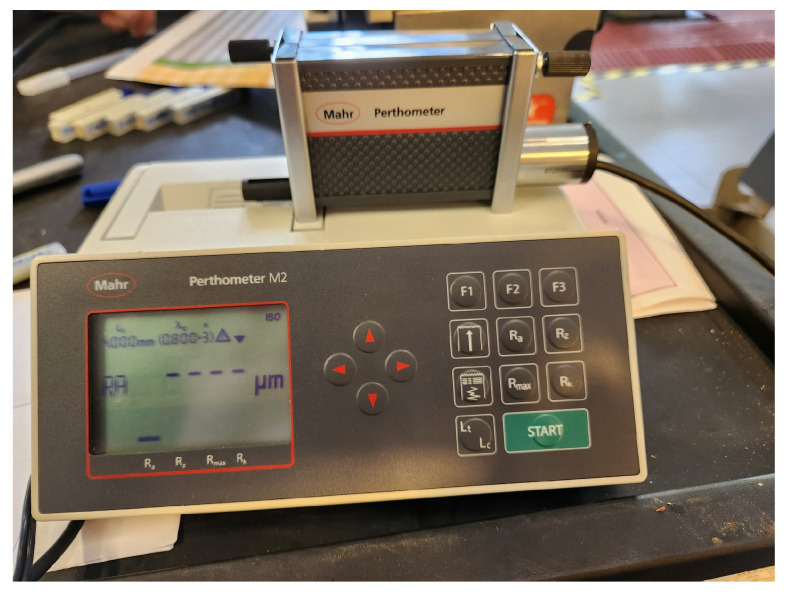
Mahr Perthometer M2 profilometer.

Regarding the roughness of the machined surface, this was measured using a Mahr Perthometer M2 profilometer (Mahr, Gottingen, Germany) (Figure 2). The test was carried out following DIN EN ISO 21920-3:2021 [57]. Each test was performed with a cut-off value (*λ*_c_) of 0.8 mm and a measurement length of 5.6 mm. Moreover, as errors may occur due to the acceleration and deceleration of the probe at the time of measurement, the first and last measurement segments of 0.8 mm were not considered. In addition, measurements were taken in the radial and tangential directions, and a minimum of five measurements were taken in different areas due to the possibility of variation in the values obtained in the centre and on the periphery of the workpiece. With these, the arithmetic average roughness value (*R*_a_) was determined.

Thus, roughness analysis was performed to evaluate the process stability and performance of the cutting tool, which can be related to TW and the best milling conditions for which it is possible to obtain the best quality and surface integrity.

#### 2.2.4. Characterisation of Wear Mechanisms

Before analysing the cutting tools’ wear, they underwent ultrasonic cleaning with acetone as a cleaning agent. Afterwards, the wear suffered by the machining tools was evaluated through Scanning Electron Microscopy (SEM) analysis, according to ISO 8688-2:1986 [58]. This standard recommends analysing the presence of all wear phenomena and adopting the one with the most significant influence as a life criterion. Thus, the VB3 was selected, and the wear measurements were performed in “Position 1”. For this, an FEI QUANTA 400 FEG scanning electron microscope was used (F.E.I., Hillsboro, OR, USA), equipped with an EDAX Genesys Energy Dispersive X-Ray Spectroscopy microanalysis system (EDAX Inc., Mahwah, NJ, USA). The analyses were carried out using BackScattered Electrons Diffraction (BSED), with magnification varying between 100× and 2000×, and using a beam potential of 15 kV, which was sporadically reduced to 10 kV.

Furthermore, Energy-Dispersive X-Ray Spectroscopy (EDS) (EDAX Inc., Mahwah, NJ, USA) analysis was used to check and confirm the occurrence of material adhered to the tool. The top view (TOP), rake face (RF), and clearance face (CF) of all tools were analysed. In addition, for better identification, a reference was created for the four cutting teeth of the tool, with numbers 1 to 4 used to identify them.

## 3. Results and Discussion

### 3.1. Roughness Analysis of the Machined Surface

**Figure 3:**
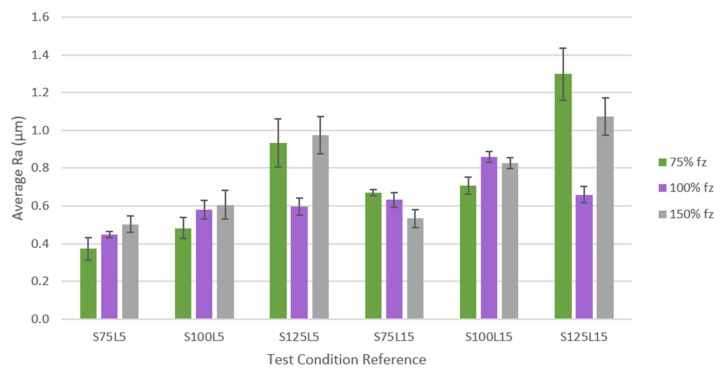
Comparison of SR values obtained for all conditions tested.

The SR was measured after each tested condition to analyse the machined surface quality in the tangential and radial directions. No notable differences were observed between the values obtained in different directions. All test conditions were compared using the SR values obtained, which were organised and grouped according to Figure 3 and are shown in Table 6. The figure is divided by test conditions in the graph’s *X* axis and three groups corresponding to each *f*_z_, as a percentage of the initial value (0.07 mm/tooth). SR values are displayed according to the *Y* axis. It should be noted that, according to the identification of the tools, the number after the “S” indicates the *v*_c_ and the number after the “L” indicates the *L*_cut_ used in the machining test.

It can be seen that by increasing the *L*_cut_, the SR values also increased, which was an already expected result, as Inconel 718 is a difficult-to-machine material that, even for small *L*_cut_ values, can generate high levels of TW, and, consequently, greater SR values and a poorer quality of the machined surface [59].

The influence of the *f*_z_ is not evident for the conditions tested at a *v*_c_ = 75 m/min since for a *L*_cut_ = 5 m, when the *f*_z_ increased, the roughness also increased. On the other hand, for a *L*_cut_ = 15 m, when the *f*_z_ was increased, the SR decreased. Usually, for lower values of *f*_z_, the quality of the machined surface is better, i.e., the roughness of the machined surface has lower values [60], meaning that the quality of the machined surface could be impaired with the increase in *f*_z_ [56]. However, for conditions with a *L*_cut_ = 15 m, this scenario was not observed since there was an improvement in the surface quality obtained. This could have been generated by stabilising the wear behaviour on the tool’s cutting edge, homogenising the wear effect on the edge.

Therefore, under the conditions tested at a *v*_c_ = 75 m/min, the maximum value of *R*_a_ was obtained for the condition that used 75% *f*_z_ and a *L*_cut_ = 15 m, i.e., for condition S75F75L15. The lowest *R*_a_ value was obtained for the condition with 75% *f*_z_ and a *L*_cut_ = 5 m, corresponding to the S75F75L5 condition. Thus, it can be seen that for 75% *f*_z_, when increasing the *L*_cut_ from 5 m to 15 m, the difference in roughness was the most notable among the tested conditions.

In the same way as for the conditions tested at 75 m/min, under the conditions tested at 100 m/min, when the *L*_cut_ was increased, the roughness also increased, making this parameter’s influence evident. Furthermore, for a *L*_cut_ = 5 m, the same was observed as in the previous case: when the *f*_z_ increased, the roughness of the machined surface also increased. However, for a *L*_cut_ = 15 m, when increasing the *f*_z_ from 75% to 100%, the roughness increased significantly, and when increasing the *f*_z_ to 150%, the roughness decreased slightly compared to 100% *f*_z_. Therefore, by increasing the *f*_z_, the roughness of the machined surface tends to be worse. Thus, it appears that this parameter greatly influences the roughness of the machined surface [61].

In the same way as in the conditions at 75 m/min and 100 m/min, in the case of using 125 m/min, when increasing the *L*_cut_ from 5 m to 15 m, the SR also increases. Regarding the *f*_z_, there seems to be some instability in the process under conditions at 75% of this parameter, given the discrepancy in the values obtained. However, for 100% and 150% of this parameter, for both 5 m and 15 m, it is observed that when the parameter increases, the roughness also increases, and the quality of the machined surface decreases.

In general, it can be seen that average *R*_a_ values tend to increase for higher *L*_cut_ values, a trend that is registered for all conditions tested. In some conditions, this increase is more pronounced, as is the case in conditions S75F75, S100F100, and S125F75. On the other hand, this increase is slight in some conditions, for example, in conditions S75F150, S125F100, and S125F150.

Regarding the *f*_z_, it is observed that this parameter also influences the roughness of the machined surface [62], but this influence is not so evident, as a common trend is not observed across all conditions. Therefore, when comparing this parameter, it is clear that for conditions S75L5 and S100L5, increasing this parameter worsens the quality of the machined surface. For the S75L15 condition, increasing this parameter results in a lower SR. On the other hand, for conditions S125L5 and S125L15, a decrease in roughness was observed when increasing from 75% of this parameter to 100%, followed by an increase in roughness when increasing to 150% of the *f*_z_. For the S100L15 condition, the opposite occurred: the roughness increased, followed by a decrease, when varying the *f*_z_. However, in general, increasing the *f*_z_ increases the roughness of the surface [63].

Furthermore, concerning the *v*_c_, it is observed that the higher this parameter, the more the roughness of the machined surface rises and the lower the surface quality. This is not commonly observed, as the tendency is for SR to decrease as this parameter increases [64]. This phenomenon probably occurred because Inconel 718 is a material that is difficult to machine, the amount of wear suffered at these *v*_c_s was high, and high abrasive wear was usually observed, which can lead to cutting tool chipping [65]. The only case in which it was observed that by increasing the *v*_c_ the *R*_a_ was lower was for 100% *f*_z_, with a *L*_cut_ = 15 m, when increasing the *v*_c_ from 100 m/min to 125 m/min.

As for standard deviation (SD), it is known that this refers to the difference in measurements at the centre and periphery of the part. In the latter, the measurement tends to be higher, as it is the end of the spiral path, and, consequently, the wear is also higher. Conditions S125F75L5 and S125F75L15 showed a more significant SD. These two conditions, as previously mentioned, suffered from instability in the process. However, this higher SD result may be related to the sustained abrasive wear of the tool, which can lead to a difference in the SR recorded from the centre to the periphery of the part [66].

For a more concise and accurate validation, *t*-tests for two samples with different variances were conducted on Microsoft^®^ Excel^™^ software to statistically assess the differences between the setups while varying a parameter, and to assess whether this parameter was the most influential when moving from one milling setup to another. A *p*-value of 0.05 was considered significant for the effects. The statistical tests started by comparing *R*_a_ results from different *L*_cut_ values and verifying the influence of this parameter while fixing *s* and *f*; for example, the *t*-test that compared the *R*_a_ from the S75F75L5 setup with the one from the S75F75L15 produced a P(T <= t) two-tailed value of 2 × 10^−4^ < 0.05, which means that there is a statistical difference between the two trials’ mean values. Table 7 summarises the aftermath of the statistical tests. All data and statistical results can be found in Appendix A.

In general, it can be said that the quality of the machined surface was satisfactory, with good SR results. The exceptions were conditions S125F75L5 and S125F75L15, which experienced instability during the process, and, consequently, their results were discrepant. One of the main characteristics of surface integrity is the presence of compressive residual stresses [67], which are generated through the HiPIMS deposition technique [68] and which also prevent cohesive failures [69]. This fact is very beneficial for producing a machined surface with low roughness and a good surface quality to the machined part [56].

### 3.2. Wear Measurements and Characterisation

**Figure 4:**
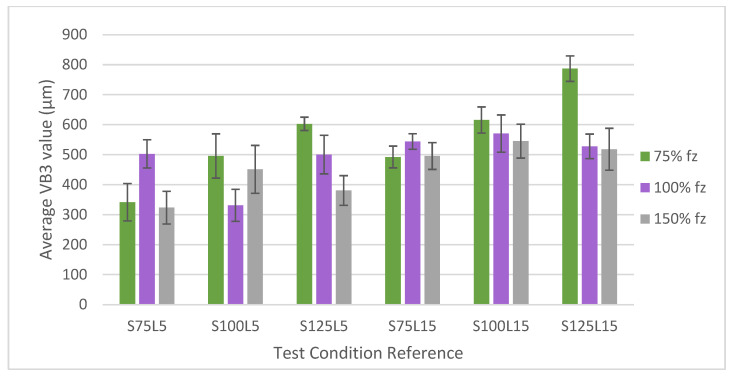
Comparison of VB3 values obtained for all conditions tested.

As explained in Section 2.2.4, the TW measurement was carried out following ISO 8688-2:1986 [58] for the top view of the tools (VB3). To compare all test conditions, the values obtained for VB3 were organised and grouped according to Figure 4; the sum is shown in Table 8. Figure 2 is divided by test conditions in the X axis of the graph, with three groups corresponding to each *f*_z_, as a percentage of the initial value (0.07 mm/tooth) in the Y axis. It should be noted that, according to the identification of the tools, the number after the “S” indicates the *v*_c_ and the number after the “L” indicates the *L*_cut_ used in the machining test.

For conditions tested at a *v*_c_ = 75 m/min, the influence of the *L*_cut_ is evidently clear when the *L*_cut_ increases from 5 m to 15 m. However, at 100% *f*_z_, this increase was slightly higher. Regarding the influence of the variation in the *f*_z_ on the flank wear (*VB* as ISO 8688-2:1986 [58] of the tools, for a *L*_cut_ = 5 m the lowest value obtained was for condition S75F150L5. Wear increased when increasing the *f*_z_ from 75% to 100% and decreased when increasing to 150%. In turn, the same trend was observed for the cases tested at a *L*_cut_ = 15 m; increased wear from 75% to 100% *f*_z_, and a reduction as the increase continued to 150%. At 100% *f*_z_, and a *L*_cut_ = 15 m, the maximum *VB* was observed. It was also noted that the condition of a *L*_cut_ = 5 m and 100% *f*_z_ presented greater wear than conditions with a *L*_cut_ = 15 m and 150% *f*_z_.

For conditions tested at 100 m/min, the same situation as in the previous case is observed regarding the *L*_cut_. When increasing from 5 m to 15 m, the resulting VB3 is higher. This is due to the properties of Inconel 718, such as the thermomechanical tool load, which generates high abrasive damage [65]. However, no clear influence of the *f*_z_ parameter is observed. For a *L*_cut_ = 5 m, when increasing from 75% to 100% *f*_z_, the VB3 decreases, and when increasing it to 150%, the VB3 increases, but with a lower value than in the first condition (F75L5). On the other hand, for a *L*_cut_ = 15 m, when the *f*_z_ is increased, the VB3 decreases in all *f*_z_ conditions.

The fact that a common trend for *f*_z_ is not observed may be related to the chip formation mechanism, which varies under each condition and is related to the productivity of the milling process [70]. It seems that the formation of thinner chips is the most common cause of a poor process performance, as they are easier to break, and this causes higher abrasion and, consequently, much wear on the cutting tool. Thus, the chip section should be thicker to lead to greater integrity and extraction flow, but if the thickness is too high it can overload the cutting edge and, consequently, breakage can occur [56].

Under the conditions tested at 125 m/min, the influence of *L*_cut_ was also observed. In all conditions, VB3 increased when *L*_cut_ increased. This is due to the properties of Inconel 718, which causes abrasive damage and can consequently lead to tool chipping [65]. However, for 100% of the *f*_z_, this increase was lessened. Furthermore, the conditions with 75% *f*_z_ presented the highest VB3, higher for 15 m than the *L*_cut_ = 5 m.

Regarding the influence of the *f*_z_ for a *L*_cut_ = 5 m, when the *f*_z_ was increased, the wear was reduced. The same occurred for a *L*_cut_ = 15 m. In other words, under these conditions, when the *L*_cut_ was increased, VB3 decreased.

In general, it can be observed that there is no clear trend for *f*_z_, making its influence more challenging to analyse and detect. As already mentioned, the *f*_z_ is directly related to the productivity of the process and the chip formation mechanism. Furthermore, Inconel 718, as a difficult-to-machine material, can generate serrated or segmented chips that affect the machined surface’s integrity [71] and result in different trends for *f*_z_.

As for the *v*_c_ used in each condition, it can be observed that when this parameter is increased in conditions with 75% *f*_z_, VB3 also increases. For conditions at 100% *f*_z_, a clear trend cannot be identified. Regarding a *L*_cut_ = 5 m and 100% *f*_z_ conditions, wear decreases when increasing from 75 m/min to 100 m/min and increases when increasing to 125 m/min. However, in the last condition, the VB3 is similar to the first condition. In turn, for 150% *f*_z_, wear increases when increasing the *v*_c_ from 75 m/min to 100 m/min and decreases when increasing to 125 m/min at both *L*_cut_ values.

Therefore, when the *v*_c_ is increased, the resulting wear also increases. This increase in *VB* is in line with the roughness obtained and the quality of the surface of the machined part. But, usually, an increased *v*_c_ results in a better surface quality and the smoother cutting behaviour of the cutting tools [72].

### 3.3. Analysis of Wear Mechanisms

#### 3.3.1. *v*_c_ of 75 m/min

**Figure 5:**
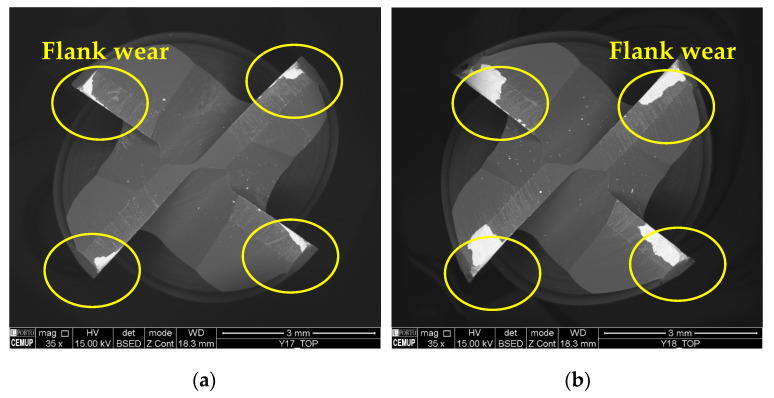
Top view of the tools tested at a *v*_c_ of 75 m/min and 35× magnification: (**a**) S75F150L5 and (**b**) S75F150L15.

Regarding the type of *VB* identified for tools tested at 75 m/min, Figure 5 illustrates the top view of the cutting tools tested under conditions S75F150L5 and S75F150L15, making it possible to observe the influence of the *L*_cut_ on the resulting wear.

**Figure 6:**
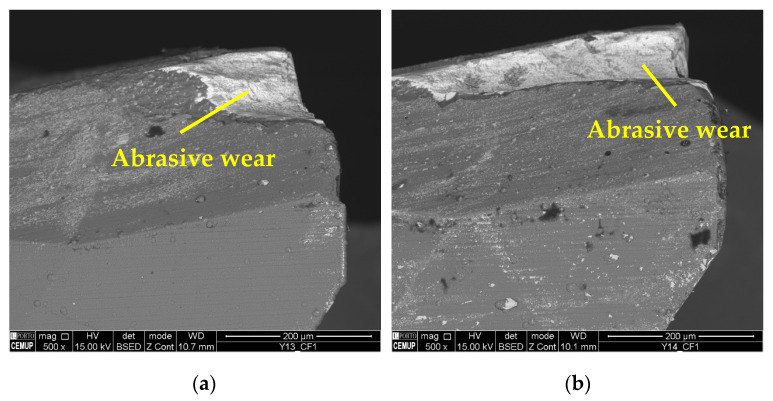
Abrasive wear: (**a**) clearance face (CF1) of S75F75L5 at 500× magnification and (**b**) clearance face (CF1) of S75F75L15 at 500× magnification.

As for the wear mechanism, it can be said that for 75 m/min conditions, mainly abrasion wear and adhesive wear were observed, both on the substrate and on the coating. In addition, in some conditions, delamination and cracking of the coating occurred. Mechanical mechanisms such as abrasion and adhesion are common in terms of the wear suffered by cutting tools in the milling process [73]. Figure 6 illustrates the abrasive wear that occurred on the substrate of the cutting tool under the S75F75L5 and S75F75L15 conditions, where, for 15 m, the wear is more developed. Abrasive wear was detected for all the tests, and was more significant and more intense for the *L*_cut_ = 15 m.

**Figure 7:**
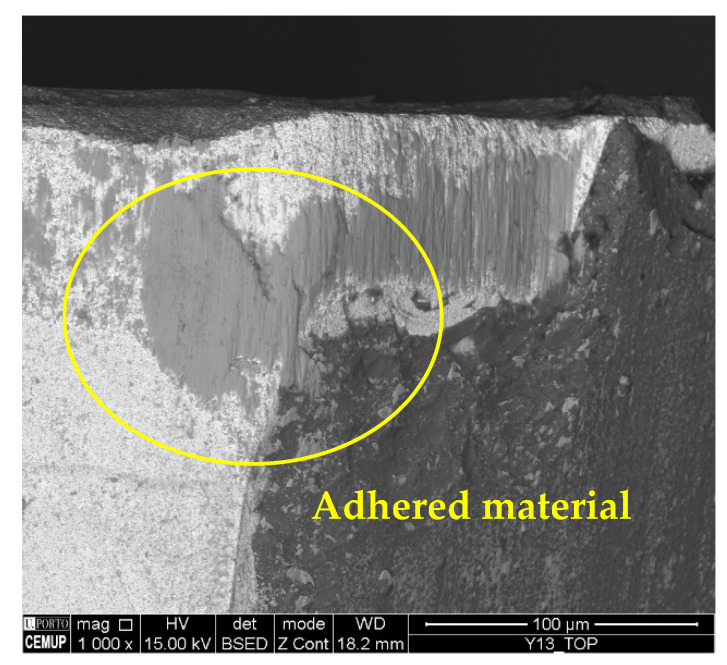
Adhered material: top view of S75F75L5 at 1000× magnification.

As for the presence of adhered material, it was found in all conditions and in great quantities. This is an expected result, since Inconel 718 usually tends to adhere to cutting tools [74]. Figure 7 illustrates the presence of adhered material on the top view of the S75F75L5 condition.

**Figure 8:**
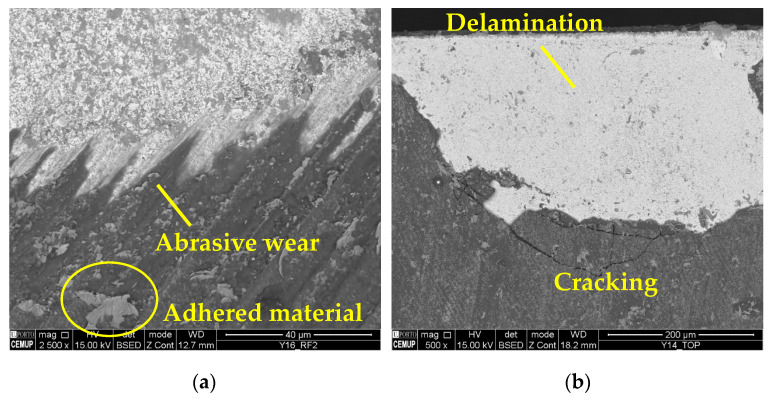
Coating wear mechanisms: (**a**) abrasive wear and adhered material in the RF2 of the S75F100L15 condition at 2500× magnification and (**b**) delamination and cracking in the top view of the S75F75L15 condition at 500× magnification.

The wear mechanisms identified in the coating were abrasive, workpiece material adhesion, delamination, and cracks. Abrasive and adhesive wear were observed at a lower intensity on the tool substrate. Material adhesion causes more abrasion and can lead to coating delamination [59]. Figure 6 illustrates the wear mechanisms identified in the coating. In Figure 8a, abrasive wear and adhered material can be observed in the S75F100L15 condition, and in Figure 8b, delamination and cracking can be seen in a cutting tool tested in the S75F75L15 condition. Furthermore, it can be stated that the wear mechanisms identified in the cutting tool’s coating were more intense under the conditions tested at the *L*_cut_ = 15 m.

Thus, it appears that the increase in *L*_cut_ caused the development of increased wear mechanisms, such as abrasion and adhesion of the material to the tool surface. It can be said that the adhesion of the material is promoted as the test progresses, as the material accumulates in the grooves left on the tool’s surface as a result of grinding [56]. Therefore, at a longer *L*_cut_, the amount of accumulated material will be higher and, consequently, more abrasive wear will occur, which can lead to coating delamination. Under these conditions, delamination occurred in almost all situations, which also indicates a low adhesion of the coating to the tool substrate. In addition, the lowest wear among all the conditions was for S75F100L5 and S75F150L5, but, as it is the condition with the lowest *v*_c_ (75 m/min), the machining process was less productive and this could increase the industrial costs.

#### 3.3.2. *v*_c_ of 100 m/min

**Figure 9:**
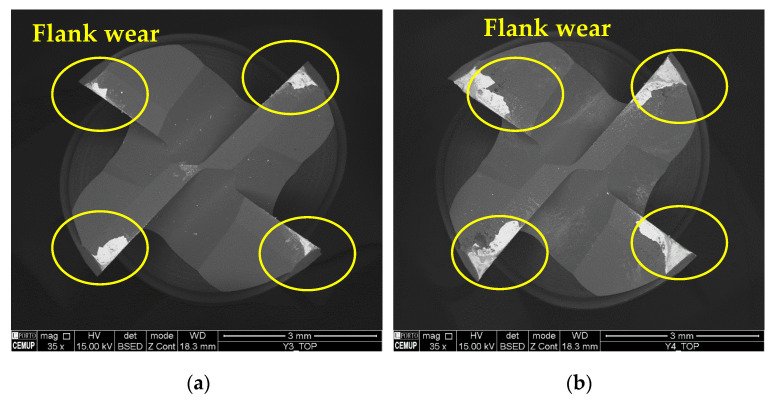
Top view of the tools tested at a *v*_c_ of 100 m/min at 35× magnification: (**a**) S100F100L5 and (**b**) S100F100L15.

Figure 9 illustrates the top view of the S100F100L5 and S100F100L15 conditions, where it is possible to observe the influence of the *L*_cut_ on the machining in these conditions.

As mentioned, the impact of the *L*_cut_ seems to significantly increase the resulting wear, as can be seen from the measurements carried out and clearly seen in the images obtained. As for the influence of *f*_z_, due to the frictional wear to which the tool is subject at lower values of this parameter [75], the wear mark is broader for tools tested with a lower *f*_z_, i.e., wear is more pronounced and deeper than in tools tested with higher values. This can modify the geometry of the tools and is also related to the chip generation process [17].

**Figure 10:**
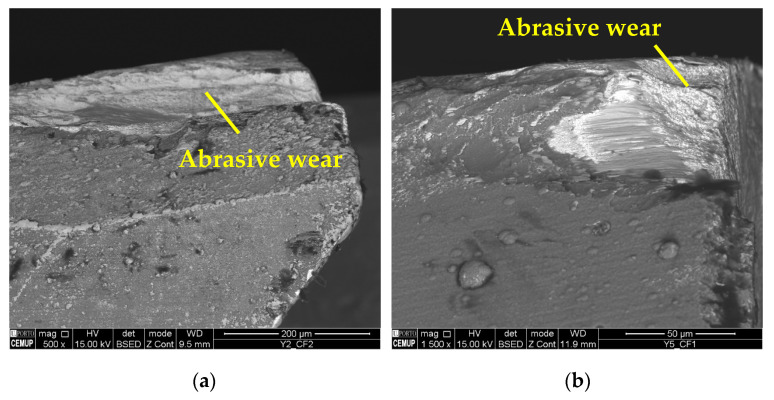
Abrasive wear: (**a**) clearance face (CF2) of S100F75L15 at 500× magnification and (**b**) clearance face (CF1) of S100F150L5 at 1500× magnification.

The predominant wear mechanisms on the tool substrate and coating were abrasion and material adhesion, the latter being more intense than in the cases tested at 75 m/min. It is seen that Inconel 718 commonly adheres to cutting tools [74]. The abrasive wear was more intense on the clearance face of the tools, as shown in Figure 10, and this type of wear is frequent when machining this alloy [76].

**Figure 11:**
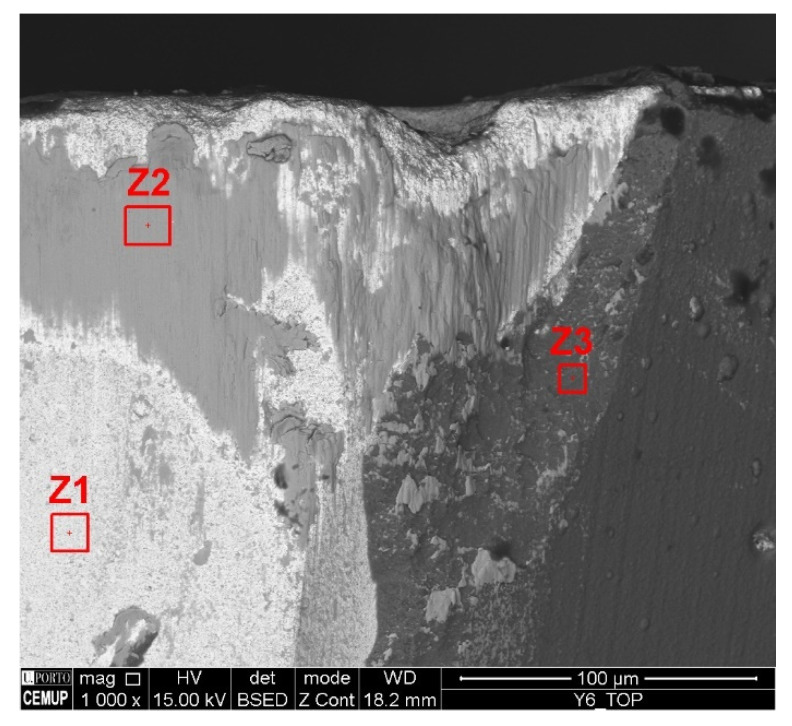
Condition S100F150L15 indicates material adhesion and has three zones for EDS analysis.

**Figure 12:**
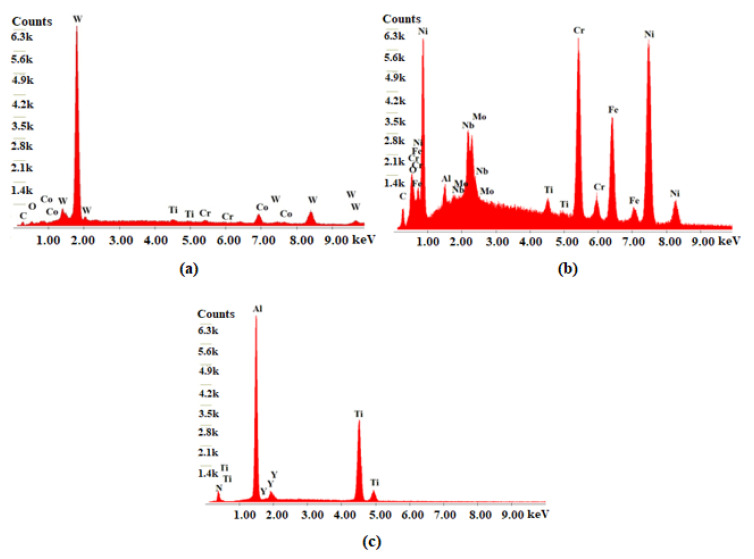
EDS spectra analysis of the three zones of S100F150L15: (**a**) Z1—tool substrate, (**b**) Z2—adhered machined material, and (**c**) Z3—coating.

Figure 11 illustrates the adhesion of the material to the tool substrate under the S100F150L15 condition, and three zones in which the analysis was carried out. This wear was registered on the tools’ flank, edges, and rake face. In this case, EDS analysis was performed to confirm that it was Inconel 718, according to the chemical composition resulting from the analysis. Figure 12 illustrates the results of the EDS analysis for the three corresponding zones.

According to the EDS analysis, zone 1, rich in tungsten, refers to the tool substrate; zone 2, in turn, is rich in Ni and with other elements present that are part of the composition of Inconel 718, indicating that it is machined material that is adhered to the substrate of the tool; and zone 3, indicating the constituent elements of the coating. Therefore, the coherence of the results obtained, based on the chemical composition of the areas indicated in the image, can be observed.

**Figure 13:**
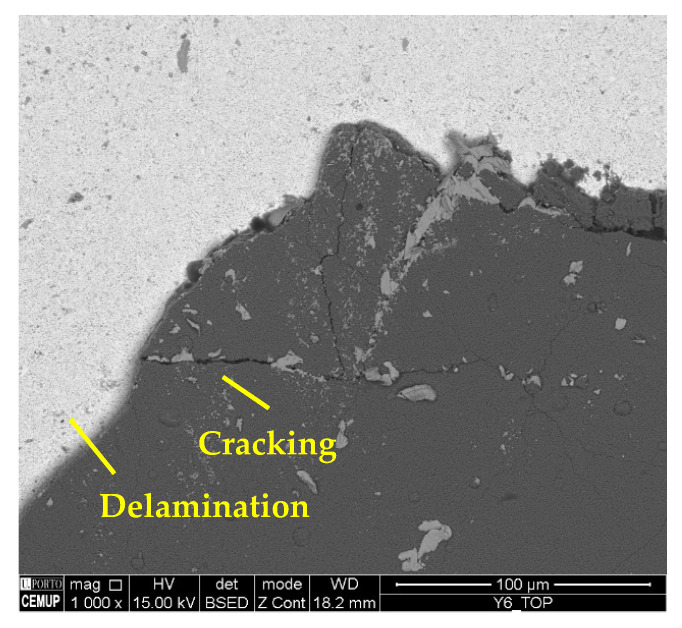
Delamination and cracking in the top view of S100F150L15 at 1000× magnification.

**Figure 14:**
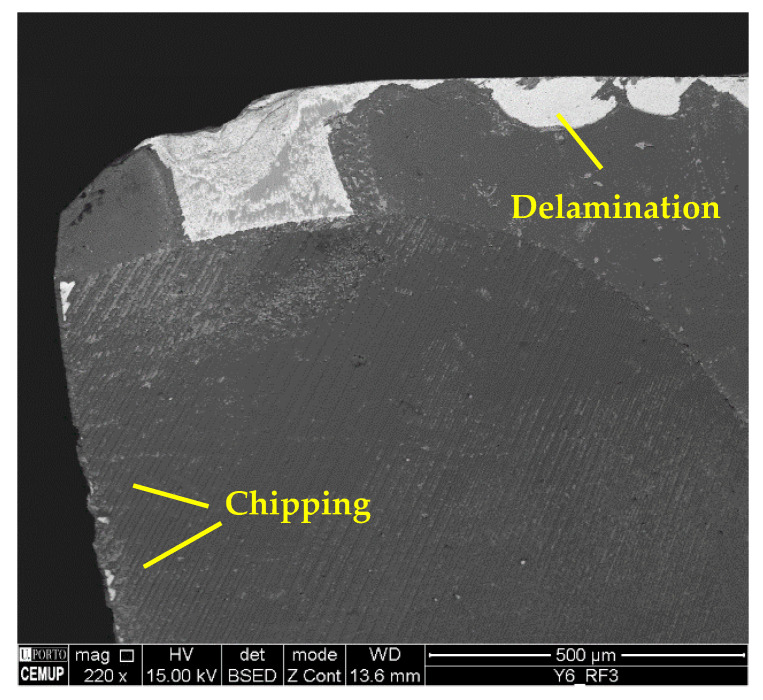
Delamination and chipping in RF3 of S100F150L15 at 220× magnification.

Regarding the wear mechanisms identified in the coating, delamination, chipping, and cracking can also be observed in addition to material adhesion and abrasion. Figure 13 illustrates cracking and delamination identified on the coating, and Figure 14 the chipping and delamination as well. This chipping may negatively affect process performance [77] due to the change in the cutting tool’s geometry [78]. Another aspect is related to cracking propagation. Some authors, such as Zhang et al. [79] and Shuai et al. [80], suggest that the presence of an interlayer could provide high strength to the coating, and that, with this, the resistance to crack propagation increases. Furthermore, thinner layers significantly increase the resistance to crack propagation and protect the coating from delamination, improving its adhesion [81,82].

#### 3.3.3. *v*_c_ of 125 m/min

**Figure 15:**
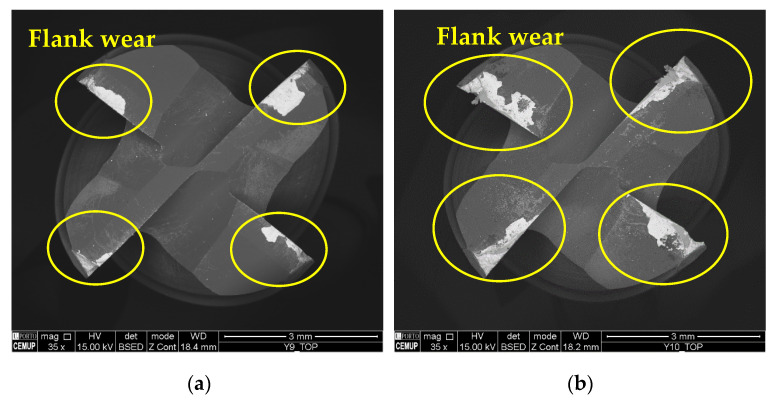
Top view of the tools tested at a *v*_c_ of 125 m/min at 35× magnification: (**a**) S125F100L5 and (**b**) S125F100L15.

Figure 15 illustrates the top view of two tools tested at 125 m/min under the S125F100L5 and S125F100L15 conditions. As seen in Section 3.3.2. the difference between these two conditions was not pronounced regarding VB3, even when the *v*_c_ increased. It can be observed that the maximum TW was similar, but in the 15 m condition it was more significant and expanded towards the centre of the cutting tool. However, in other conditions, as expected, the severity of the wear increased for higher values of *L*_cut_, which further intensified the wear phenomena.

**Figure 16:**
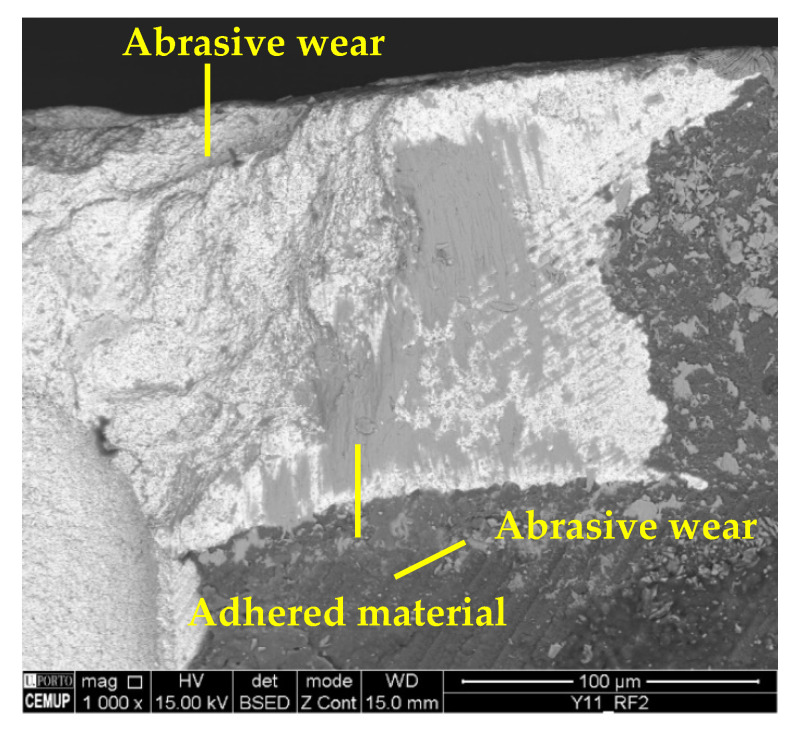
Wear mechanisms in condition S125F150L5: abrasive and adhesive wear on the tool substrate and coating.

**Figure 17:**
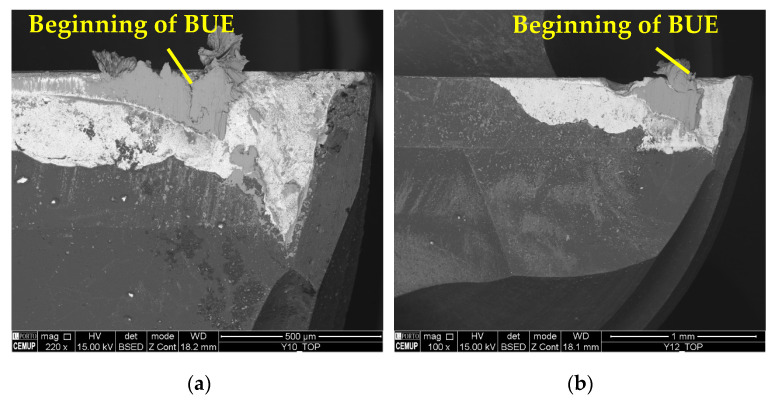
Beginning of BUE development: (**a**) top view of S125F100L15 with 220× magnification and (**b**) top view of S125F150L15 with 100× magnification.

As in previous cases, the main wear mechanisms observed were abrasion and adhesion. However, under these conditions, the wear was higher and more expressive, with the beginning of the development of a BUE under the conditions tested at a *L*_cut_ = 15 m. The beginning of the development of a BUE indicates a large amount of adhered material, which tends to generate severe wear [83], as this mechanism increases abrasive wear and, consequently, leads to the occurrence of the delamination of the coating. Figure 16 illustrates the abrasive wear and material adhesion, both on the coating and the tool substrate, and Figure 17 illustrates the beginning of BUE development.

**Figure 18:**
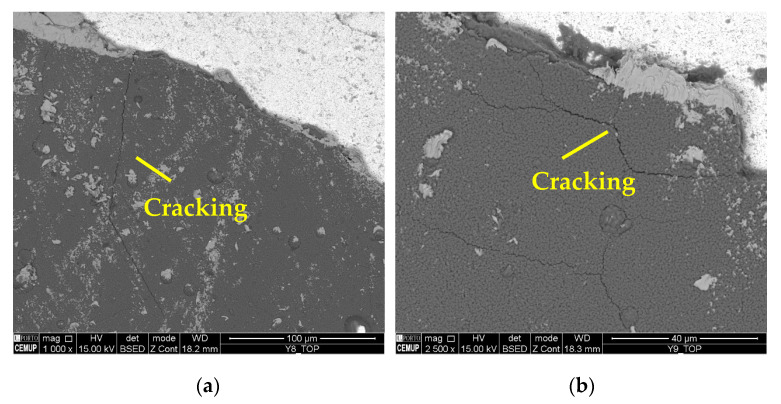
Cracking in the coating: (**a**) top view of S125F75L15 at 1000× magnification and (**b**) top view of S125F100L5 at 2500× magnification.

**Figure 19:**
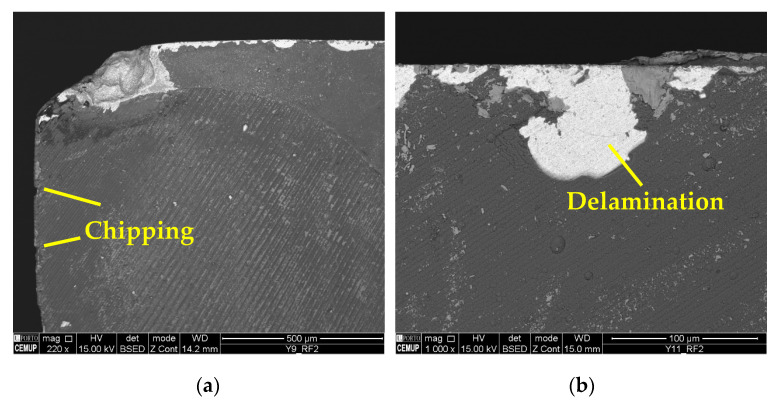
Coating wear mechanisms: (**a**) chipping in RF2 of S125F100L5 condition at 220× magnification and (**b**) delamination in RF2 of S125F150L5 condition at 1000× magnification.

It should be noted that the BUE is more common at a lower *v*_c_, and changes the tool’s geometry, which can accelerate TW [59]. The machining of Inconel 718 can be reasonably aggressive on cutting tools, promoting high levels of wear [76]. Furthermore, cracking, chipping, and delamination were observed in the coating. The formation of chips impairs the performance of the machining process, as this type of wear changes the geometry of the cutting tools, which causes the chip generation mechanisms to be modified, and consequently, the quality of the product obtained is lower [84]. Figure 18 illustrates the cracks in the coating seen under the S125F75L15 and S125F100L5 conditions, and Figure 19 illustrates chipping (S125F100L5) and delamination (S125F150L5).

From the wear mechanisms identified, the difficulty of milling operations with Inconel 718 can be seen. But not only Inconel 718 brings this difficulty, which results in severe wear mechanisms; authors such as Martinho et al. [85] have analysed and detected similar mechanisms as a result of the machining of other materials that are also considered difficult to machine.

## 4. Conclusions

The present work describes a comparative evaluation of milling parameters regarding their influence on the quality of the machined surface and the resulting tool wear. Machining parameters such as *v*_c_, *f*_z_, and *L*_cut_ were altered for this evaluation. Regarding the results obtained, the following conclusions can be drawn:

- The machining parameters influence the process, with the *L*_cut_ having the highest influence;
- The lowest roughness values for the machined surface were obtained using the S75F75L5 condition;
- Due to sustained TW, the *v*_c_ had no apparent influence on roughness;
- Regarding the SD observed in SR measurements, it can be stated that the change in the measurements from the centre to the periphery of different conditions was induced by the increased TW observed during the path followed by the tool for the machining strategy adopted;
- The greatest *VB* was observed for the S125F75L15 condition, and the lowest for the S75F150L5 condition, which were clearly influenced by the *L*_cut_;
- For higher *v*_c_ and *L*_cut_ values, the wear developed was more intense;
- The predominant wear mechanisms were abrasive and adhesive wear on the coating and the tool substrate. Delamination, chipping, and cracking were also observed in the coated tools;
- At a *L*_cut_ of 15 m and *v*_c_ of 125 m/min, BUE development was generated;
- Even at a *L*_cut_ = 5 m, much wear was observed, which indicates the difficulty of machining Inconel 718, leading to the realisation that these cutting tools are unsuitable for these kinds of operations with higher *L*_cut_ values.

Therefore, the results show a need to improve the process further, especially regarding the high wear on the coating. The coatings’ adhesion must also be improved, since coating delamination was observed under all conditions. Therefore, adding an interlayer before applying the coating now used is suggested, with the aim to improve its adhesion and reduce cracking propagation, consequently improving the process performance. Furthermore, the wear behaviour of this coating in cutting tools of different geometries can be compared with the results obtained in this work. Moreover, the results clearly indicate the need for a new study focused on the coating deposition parameters and the optimisation of machining parameters.

In addition, as a limitation of this study, the difficulty of machining Inconel 718 is highlighted, as well as the use of few machining conditions, making it necessary to expand them so that this analysis would be more complete. Based on this, for future work we recommend the use of more samples and machining conditions, as well as the use of a interlayer coating in order to avoid delamination, and the comparison of this coating’s behaviour with other coatings and with uncoated tools. In addition, we recommend carrying out a statistical analysis of the results obtained.

## Figures and Tables

**Table 1:** Mechanical properties of the Ni superalloy Inconel 718.

Material Property	Value
Yield strength [MPa]	1200
Tensile strength [MPa]	1427
Hardness [HBW]	441

**Table 2:** Chemical composition of Inconel 718 (wt%) [56].

Elements (%wt)							
Ni	Cr	Fe	Nb	Mo	Ti	Al	Co
53.89	18.05	17.78	5.35	2.90	0.96	0.51	0.20
Cu	Si	Mg	B	C	P	N	Mg
0.10	0.08	0.078	0.039	0.023	0.010	0.007	0.0017

**Table 3:** The geometry of the WC-Co end mills used in the experimental work.

Tool Geometry	Dimensions
Cutting Ø	6 mm
Total length	57 mm
Maximum cutting depth	13 mm
Number of flutes	4
Rake angle	12°
Clearance angle	10°
Chamfer	45°; 0.20 mm
Helix angle	35°

**Table 4:** Parameters of the deposition of TiAlYN coating.

Deposition Parameters	TiAlYN Layer
Reactor gases	Ar^+^ + Kr + N_2_
Deposition time [min]	233
Target amount/composition	4/TiAlY
Pressure [mPa]	600
Bias voltage [V]	−60
Temperature [°C]	520
Holder rotational speed [rpm]	1

**Table 5:** Parameters and conditions used in milling tests.

Reference	*v*_c_ [m/min]	*f*_z_ [mm/tooth]	*L*_cut_ [m]	*a*_p_ [mm]	*a*_e_ [mm]	*T* [min]
S75F75L5	75.0000	0.0525	5.0000	0.0800	4.5000	5.9854
S75F75L15	75.0000	0.0525	15.0000	0.0800	4.5000	17.9563
S75F100L5	75.0000	0.0700	5.0000	0.0800	4.5000	4.4880
S75F100L15	75.0000	0.0700	15.0000	0.0800	4.5000	13.4640
S75F150L5	75.0000	0.1050	5.0000	0.0800	4.5000	2.9919
S75F150L15	75.0000	0.1050	15.0000	0.0800	4.5000	8.9759
S100F75L5	100.0000	0.0525	5.0000	0.0800	4.5000	4.4880
S100F75L15	100.0000	0.0525	15.0000	0.0800	4.5000	13.4639
S100F100L5	100.0000	0.0700	5.0000	0.0800	4.5000	3.3659
S100F100L15	100.0000	0.0700	15.0000	0.0800	4.5000	10.0978
S100F150L5	100.0000	0.1050	5.0000	0.0800	4.5000	2.2440
S100F150L15	100.0000	0.1050	15.0000	0.0800	4.5000	6.7319
S125F75L5	125.0000	0.0525	5.0000	0.0800	4.5000	3.5904
S125F75L15	125.0000	0.0525	15.0000	0.0800	4.5000	10.7711
S125F100L5	125.0000	0.0700	5.0000	0.0800	4.5000	2.6928
S125F100L15	125.0000	0.0700	15.0000	0.0800	4.5000	8.0783
S125F150L5	125.0000	0.1050	5.0000	0.0800	4.5000	1.7952
S125F150L15	125.0000	0.1050	15.0000	0.0800	4.5000	5.3856

**Table 6:** *R*_a_ values for all conditions tested.

Reference	Average *R*_a_ Value (µm)
S75F75L5	0.372 ± 0.0060
S75F75L15	0.670 ± 0.0155
S75F100L5	0.448 ± 0.0176
S75F100L15	0.631 ± 0.0382
S75F150L5	0.502 ± 0.0434
S75F150L15	0.533 ± 0.0472
S100F75L5	0.483 ± 0.0542
S100F75L15	0.708 ± 0.0444
S100F100L5	0.578 ± 0.0493
S100F100L15	0.859 ± 0.0301
S100F150L5	0.605 ± 0.0755
S100F150L15	0.827 ± 0.0279
S125F75L5	0.935 ± 0.1270
S125F75L15	1.299 ± 0.2759
S125F100L5	0.595 ± 0.0454
S125F100L15	0.659 ± 0.0431
S125F150L5	0.975 ± 0.0988
S125F150L15	1.073 ± 0.0999

**Table 7:** Drawn conclusions from the performed *t*-tests.

Condition	Comments
*L*_cut_ influence	For low values of *s* and *f*, *L*_cut_ has the most influence. However, as the values of *s* and *f* increase, its influence on the surface quality becomes less prominent. In the case of S75F150L5 vs. S75F150L15, surface quality is not affected by the *L*_cut_. For cases with conditions S100F150L15 and above, there is a noticeable blend of the influence of all three parameters together.
*f* influence	*f* is the most influential parameter on the surface quality and this influence is more pronounced when accompanied by an increasing in *s* within the setup. However, due to phenomena such as three-body abrasion, some outliers are to be seen, since the milling setup has proven to be catastrophic to TiAlYN-coated tools.
*s* influence	*s* has proven to be the most sensitive parameter, when in conjunction with *f* and *L*_cut_. In some cases, it is visible that the increase in *s* leads to a different variance in the measured values, but sometimes it does not. Nonetheless, for the most extreme setups carried out, *s* is a very influential parameter regarding surface quality.

**Table 8:** Average values of VB3 for all conditions tested.

Reference	Average VB3 Value (µm)
S75F75L5	341.67 ± 124.349
S75F75L15	492.15 ± 72.7340
S75F100L5	502.68 ± 94.3731
S75F100L15	543.75 ± 25.8692
S75F150L5	323.43 ± 109.361
S75F150L15	495.38 ± 89.2471
S100F75L5	495.77 ± 147.663
S100F75L15	615.55 ± 43.5210
S100F100L5	331.09 ± 106.354
S100F100L15	570.41 ± 62.1473
S100F150L5	450.84 ± 79.8641
S100F150L15	545.21 ± 56.4712
S125F75L5	602.74 ± 22.1942
S125F75L15	786.95 ± 85.0253
S125F100L5	500.13 ± 128.609
S125F100L15	527.68 ± 41.0967
S125F150L5	380.68 ± 98.4872
S125F150L15	518.06 ± 70.0723

## Data Availability

No new data were created.

## Appendix A

Here lies the statistical analysis on the influence of each parameter on surface quality.

**Table A1:** *t*-test: two samples with different variances, comparison between their *L*_cut_ values, part 1.

	S75F75L5	S75F75L15	S75F100L5	S75F100L15	S75F150L5	S75F150L15	S100F75L5	S100F75L15	S100F100L5	S100F100L15	S100F150L5	S100F150L15
Mean	0.372	0.670	0.448	0.631	0.502	0.533	0.483	0.708	0.578	0.859	0.605	0.827
Variance	0.005	0.000	0.000	0.002	0.002	0.003	0.004	0.002	0.003	0.001	0.007	0.001
Observations	5	5	5	5	5	5	5	5	5	5	5	5
Hypothesised mean Difference	0		0		0		0		0		0	
df	5		6		8		8		7		5	
t Stat	−9.612		−8.683		−0.954		−6.430		−9.728		−5.501	
P(T <= t) one-tail	0.000		0.000		0.184		0.000		0.000		0.001	
t critical one-tail	2.015		1.943		1.860		1.860		1.895		2.015	
P(T <= t) two-tail	0.000		0.000		0.368		0.000		0.000		0.003	
t critical two-tail	2.571		2.447		2.306		2.306		2.365		2.571	

**Table A2:** *t*-Test: two samples with different variances, comparison between their *L*_cut_ values, part 2.

	S125F75L5	S125F75L15	S125F100L5	S125F100L15	S125F150L5	S125F150L15
Mean	0.935	1.299	0.595	0.659	0.975	1.073
Variance	0.020	0.095	0.003	0.002	0.012	0.012
Observations	5	5	5	5	5	5
Hypothesised mean Difference	0		0		0	
df	6		8		8	
t Stat	−2.398		−2.039		−1.392	
P(T <= t) one-tail	0.027		0.038		0.101	
t critical one-tail	1.943		1.860		1.860	
P(T <= t) two-tail	0.053		0.076		0.201	
t critical two-tail	2.447		2.306		2.306	

**Table A3:** *t*-Test: two samples with different variances, comparison between their *f* values, part 1.

	S75F75L5	S75F100L5	S75F75L15	S75F100L15	S100F75L5	S100F100L5	S100F75L15	S100F100L15	S125F75L5	S125F100L5	S125F75L15	S125F100L15
Mean	0.372	0.448	0.670	0.631	0.483	0.578	0.708	0.859	0.935	0.595	1.299	0.659
Variance	0.005	0.000	0.000	0.002	0.004	0.003	0.002	0.001	0.020	0.003	0.095	0.002
Observations	5	5	5	5	5	5	5	5	5	5	5	5
Hypothesised Mean Difference	0		0		0		0		0		0	
df	5		5		8		7		5		4	
t Stat	−2.435		1.891		−2.592		−5.614		5.036		4.584	
P(T <= t) one-tail	0.029		0.059		0.016		0.000		0.002		0.005	
t critical one-tail	2.015		2.015		1.860		1.895		2.015		2.132	
P(T <= t) two-tail	0.059		0.117		0.032		0.001		0.004		0.010	
t critical two-tail	2.571		2.571		2.306		2.365		2.571		2.776	

**Table A4:** *t*-Test: two samples with different variances, comparison between their *f* values, part 2.

	S75F100L5	S75F150L5	S75F100L15	S75F150L15	S100F100L5	S100F150L5	S100F100L15	S100F150L15	S125F100L5	S125F150L5	S125F100L15	S125F150L15
Mean	0.448	0.502	0.631	0.533	0.578	0.605	0.859	0.827	0.595	0.975	0.659	1.073
Variance	0.000	0.002	0.002	0.003	0.003	0.007	0.001	0.001	0.003	0.012	0.002	0.012
Observations	5	5	5	5	5	5	5	5	5	5	5	5
Hypothesised Mean Difference	0		0		0		0		0		0	
df	5		8		7		8		6		5	
t Stat	−2.295		3.238		−0.608		1.571		−6.987		−7.607	
P(T <= t) one-tail	0.035		0.006		0.281		0.077		0.000		0.000	
t critical one-tail	2.015		1.860		1.895		1.860		1.943		2.015	
P(T <= t) two-tail	0.070		0.012		0.563		0.155		0.000		0.001	
t critical two-tail	2.571		2.306		2.365		2.306		2.447		2.571	

**Table A5:** *t*-Test: two samples with different variances, comparison between their *f* values, part 3.

	S75F75L5	S75F150L5	S75F75L15	S75F150L15	S100F75L5	S100F150L5	S100F75L15	S100F150L15	S125F75L5	S125F150L5	S125F75L15	S125F150L15
Mean	0.372	0.502	0.670	0.533	0.483	0.605	0.708	0.827	0.935	0.975	1.299	1.073
Variance	0.005	0.002	0.000	0.003	0.004	0.007	0.002	0.001	0.020	0.012	0.095	0.012
Observations	5	5	5	5	5	5	5	5	5	5	5	5
Hypothesised Mean Difference	0		0		0		0		0		0	
df	7		5		7		7		8		5	
t Stat	−3.509		5.527		−2.633		−4.514		−0.500		1.542	
P(T <= t) one-tail	0.005		0.001		0.017		0.001		0.315		0.092	
t critical one-tail	1.895		2.015		1.895		1.895		1.860		2.015	
P(T <= t) two-tail	0.010		0.003		0.034		0.003		0.631		0.184	
t critical two-tail	2.365		2.571		2.365		2.365		2.306		2.571	

**Table A6:** *t*-Test: two samples with different variances, comparison between their *s* values, part 1.

	S75F75L5	S100F75L5	S75F75L15	S100F75L15	S75F100L5	S100F100L5	S75F100L15	S100F100L15	S75F150L5	S100F150L5	S75F150L15	S100F150L15
Mean	0.372	0.483	0.670	0.708	0.448	0.578	0.631	0.859	0.502	0.605	0.533	0.827
Variance	0.005	0.004	0.000	0.002	0.000	0.003	0.002	0.001	0.002	0.007	0.003	0.001
Observations	5	5	5	5	5	5	5	5	5	5	5	5
Hypothesised Mean Difference	0		0		0		0		0		0	
df	8		5		5		8		6		6	
t Stat	−2.739		−1.623		−4.948		−9.368		−2.369		−10.72	
P(T <= t) one-tail	0.013		0.083		0.002		0.000		0.028		0.000	
t critical one-tail	1.860		2.015		2.015		1.860		1.943		1.943	
P(T <= t) two-tail	0.025		0.165		0.004		0.000		0.056		0.000	
t critical two-tail	2.306		2.571		2.571		2.306		2.447		2.447	

**Table A7:** *t*-Test: two samples with different variances, comparison between their *s* values, part 2.

	S100F75L5	S125F75L5	S100F75L15	S125F75L15	S100F100L5	S125F100L5	S100F100L15	S125F100L15	S100F150L5	S125F150L5	S100F150L15	S125F150L15
Mean	0.483	0.935	0.708	1.299	0.578	0.595	0.859	0.659	0.605	0.975	0.827	1.073
Variance	0.004	0.020	0.002	0.095	0.003	0.003	0.001	0.002	0.007	0.012	0.001	0.012
Observations	5	5	5	5	5	5	5	5	5	5	5	5
Hypothesised Mean Difference	0		0		0		0		0		0	
df	5		4		8		7		7		5	
t Stat	−6.543		−4.227		−0.513		7.611		−5.945		−4.744	
P(T <= t) one-tail	0.001		0.007		0.311		0.000		0.000		0.003	
t critical one-tail	2.015		2.132		1.860		1.895		1.895		2.015	
P(T <= t) two-tail	0.001		0.013		0.622		0.000		0.001		0.005	
t critical two-tail	2.571		2.776		2.306		2.365		2.365		2.571	

**Table A8:** *t*-Test: two samples with different variances, comparison between their *s* values, part 3.

	S75F75L5	S125F75L5	S75F75L15	S125F75L15	S75F100L5	S125F100L5	S75F100L15	S125F100L15	S75F150L5	S125F150L5	S75F150L15	S125F150L15
Mean	0.372	0.935	0.670	1.299	0.448	0.595	0.631	0.659	0.502	0.975	0.533	1.073
Variance	0.005	0.020	0.000	0.095	0.000	0.003	0.002	0.002	0.002	0.012	0.003	0.012
Observations	5	5	5	5	5	5	5	5	5	5	5	5
Hypothesised Mean Difference	0		0		0		0		0		0	
df	6		4		5		8		5		6	
t Stat	−8.009		−4.551		−6.031		−0.965		−8.762		−9.774	
P(T <= t) one-tail	0.000		0.005		0.001		0.181		0.000		0.000	
t critical one-tail	1.943		2.132		2.015		1.860		2.015		1.943	
P(T <= t) two-tail	0.000		0.010		0.002		0.363		0.000		0.000	
t critical two-tail	2.447		2.776		2.571		2.306		2.571		2.447	
